# The use of semaglutide as an add-on therapy in patients with latent autoimmune diabetes in adults

**DOI:** 10.1210/clinem/dgag072

**Published:** 2026-02-23

**Authors:** Maria Elena Lunati, Vincenzo Cimino, Davide Bernasconi, Cristina Romano, Olga Disoteo, Antonio Rossi, Camilla Tinari, Roberta Maria Fiorina, Alessandra Gandolfi, Paola Silvia Morpurgo, Francesca D’Addio, Elisa Lazzaroni, Fabrizio Losurdo, Ida Pastore, Laura Molteni, Cesare Berra, Moufida Ben Nasr, Laura Montefusco, Loredana Bucciarelli, Paolo Fiorina

**Affiliations:** Division of Endocrinology, ASST Fatebenefratelli-Sacco, 20121 Milan, Italy; Division of Endocrinology, ASST Fatebenefratelli-Sacco, 20121 Milan, Italy; Department of Biomedical and Clinical Sciences, University of Milan, 20157 Milan, Italy; Division of Endocrinology, ASST Fatebenefratelli-Sacco, 20121 Milan, Italy; Diabetology, Azienda Ospedaliera ASST Sette Laghi - Osp. di Circolo, 21100 Varese, Italy; Division of Endocrinology and Diabetology, Sant'Anna Hospital - ASST Lariana, 22100 Como, Italy; Department of Biomedical and Clinical Sciences, University of Milan, 20157 Milan, Italy; Department of Biomedical and Clinical Sciences, University of Milano, 20157 Milan, Italy; IRCCS Ospedale Galeazzi-Sant'Ambrogio, 20157 Milano, Italy; Division of Endocrinology, ASST Fatebenefratelli-Sacco, 20121 Milan, Italy; International Center for T1D, Pediatric Clinical Research Center Romeo ed Enrica Invernizzi, DIBIC, University of Milan, 20157 Milan, Italy; Division of Endocrinology, ASST Fatebenefratelli-Sacco, 20121 Milan, Italy; Division of Endocrinology, ASST Fatebenefratelli-Sacco, 20121 Milan, Italy; Division of Endocrinology, ASST Fatebenefratelli-Sacco, 20121 Milan, Italy; Department of Biomedical and Clinical Sciences, University of Milan, 20157 Milan, Italy; International Center for T1D, Pediatric Clinical Research Center Romeo ed Enrica Invernizzi, DIBIC, University of Milan, 20157 Milan, Italy; Division of Endocrinology, ASST Fatebenefratelli-Sacco, 20121 Milan, Italy; Division of Endocrinology, ASST Fatebenefratelli-Sacco, 20121 Milan, Italy; Department of Biomedical and Clinical Sciences, University of Milan, 20157 Milan, Italy; Division of Endocrinology, ASST Fatebenefratelli-Sacco, 20121 Milan, Italy; Department of Biomedical and Clinical Sciences, University of Milan, 20157 Milan, Italy; Centre for Diabetology, Endocrinology and Treatment of Metabolic Diseases, Sacra Famiglia Hospital, 22036 Erba, Italy; Department of Endocrinology and Metabolic Diseases, IRCCS Multimedica, 20099 Milan, Italy; Department of Biomedical and Clinical Sciences, University of Milan, 20157 Milan, Italy; International Center for T1D, Pediatric Clinical Research Center Romeo ed Enrica Invernizzi, DIBIC, University of Milan, 20157 Milan, Italy; Nephrology Division, Boston Children's Hospital, Harvard Medical School, Boston, MA 02115, USA; Division of Endocrinology, ASST Fatebenefratelli-Sacco, 20121 Milan, Italy; Department of Biomedical and Clinical Sciences, University of Milan, 20157 Milan, Italy; International Center for T1D, Pediatric Clinical Research Center Romeo ed Enrica Invernizzi, DIBIC, University of Milan, 20157 Milan, Italy; Pio Albergo Trivulzio, 20146 Milan, Italy; International Center for T1D, Pediatric Clinical Research Center Romeo ed Enrica Invernizzi, DIBIC, Università di Milano, 20146 Milan, Italy; Division of Endocrinology, ASST Fatebenefratelli-Sacco, 20121 Milan, Italy; Department of Biomedical and Clinical Sciences, University of Milan, 20157 Milan, Italy; International Center for T1D, Pediatric Clinical Research Center Romeo ed Enrica Invernizzi, DIBIC, University of Milan, 20157 Milan, Italy; Nephrology Division, Boston Children's Hospital, Harvard Medical School, Boston, MA 02115, USA

**Keywords:** GLP-1RA, LADA, semaglutide, β-cell

## Abstract

**Introduction:**

Semaglutide, a GLP-1R agonist (GLP-1RA), has demonstrated high efficacy in the management of type 2 diabetes. Few data in literature are available regarding the use of this agent in patients affected by latent autoimmune diabetes in adults (LADA). The purpose of this study is to analyze the efficacy of semaglutide use in patients affected by LADA.

**Materials and methods:**

In this retrospective study, we collected and analyzed data of 80 patients with LADA treated with semaglutide, either oral or subcutaneous, as an add-on therapy to insulin. Laboratory and clinical parameters and metrics from continuous glucose monitoring were collected where available.

**Results:**

Among 80 patients, 68 continued semaglutide (57/68 oral and 11/68 subcutaneous) for at least 6 months, whereas 12/80 patients discontinued treatment. After 6 months, semaglutide users showed statistically significant reduction in glycated hemoglobin, body mass index, and insulin total daily dose. Interestingly, serum C-peptide levels and the time-in-range values increased, without changes in the time below range. Subjects with residual β-cell function showed a higher body mass index and insulin total daily dose reduction. Moreover, the subgroup of subjects with preserved β-cell mass showed a greater improvement in time in range as compared to those with poor C-peptide production. Finally, 14/68 subjects suspended insulin bolus administration after starting semaglutide.

**Conclusion:**

Semaglutide as an add-on treatment to insulin exerted relevant clinical beneficial effects on the glycometabolic control in patients with LADA. These effects are enhanced in those patients with preserved β-cell function.

Latent autoimmune diabetes in adults (LADA), first described in 1993, is a subclass of patients with intermediate characteristics between type 1 diabetes (T1D) and type 2 diabetes (T2D) and accounts for 2% to 12% of all patients with diabetes (1, 2). The main criteria for the diagnosis of LADA, as suggested by the Immunology of Diabetes Society, consist mainly in a late age of presentation (nearly 30 years) with a slow evolution toward a complete loss of β-cell function (3). LADA can be considered a border between the 2 main types of diabetes for its halfway phenotypic features (4), with high heterogeneity and a mixed phenotype (4). Patients with LADA show a highly variable rate of β-cell destruction, much more heterogeneous than young-onset autoimmune diabetes, which is influenced by glutamic acid decarboxylase antibody (GADA) titer, 2 or more positives for diabetes-associated autoantibodies (5), the presence of genetic factors, and a differential and inter-individual severity of the autoimmune process (6). In addition to insulin deficiency, patients with LADA also showed clinical features of insulin resistance and obesity, resembling T2D (7). Given this heterogeneity of LADA, insulin is not the only therapeutic option. Much more insight has been conferred to GLP-1R agonists because of their pleiotropic action and effectiveness in reducing cardiovascular risk while maintaining a low hypoglycemic risk (8, 9), which could be an attractive therapy for the management of patients with LADA, particularly in those with preserved β-cell function (10). The evaluation of C-peptide levels is therefore necessary to determine proper clinical management strategies (7, 10). Many lines of the literature regarding the use of GLP-1R agonists in patients with LADA derive from retrospective analyses or post hoc analyses. A post hoc analysis of the AWARD studies demonstrated the comparable efficacy of dulaglutide in both GADA-positive and GADA-negative patients (11). Despite GLP-1R agonists still not being approved for the treatment of autoimmune diabetes, their off-label use is growing exponentially. The aim of this study is to analyze the short-term effects of semaglutide on the glycometabolic parameters and weight control in a population of patients affected by LADA, secondarily to evaluate variations in glucose metrics and insulin daily dose, to provide a better insight into the role of GLP-1R agonists in LADA patients.

## Materials and methods

### Trial design and participants

We aimed to investigate the effects of semaglutide treatment (oral or subcutaneous) in patients affected by LADA in several diabetes centers in Lombardia, Italy. The participating centers were: Sacco, Melloni and Fatebenefratelli Hospitals (Milan), Multimedica Sesto San Giovanni Hospital, Ospedale di Circolo di Varese, Melegnano Hospital, and Sacra Famiglia Hospital (Como). Only patients with a confirmed diagnosis of LADA according to the expert consensus statement (10), who received semaglutide based on independent clinical choice were included. The diagnosis of LADA was made based on the 3 criteria defined by the Immunology for Diabetes: (1) age greater than 30 years; (2) positive autoantibodies to islet β cells; and (3) insulin independence for at least 6 months after initial diagnosis. Antibodies were tested via hospital laboratory using an ELISA-based anti-glutamic acid decarboxylase antibody (EUROIMMUN, catalog no. EA 1022-9601G, RRID: AB_3739755) and chemiluminescent immunoassay (Shenzhen New Industries Biomedical Engineering, catalog no. 130205005M, RRID: AB_3739761), an ELISA-based anti-IA-2 antibody (EUROIMMUN, catalog no. EA 1023-9601G, RRID: AB_3739756), an ELISA-based anti-insulin antibody (ORGENTEC, catalog no. ORG 520, RRID: AB_2889858), an ELISA-based anti-ZnT8 antibody (EUROIMMUN, catalog no. EA 1027-9601, RRID: AB_3073786), and islet cell antibodies indirect immunofluorescence assay (EUROIMMUN, catalog no. CA 1021-0502, RRID: AB_3739758). Written informed consent was obtained from each participant, and the study was approved by the Local Ethics Committee. Every clinician involved in the study followed insulin titration as suggested by the American Diabetes Association standard of care (1). Basal insulin was titrated every 2 to 3 days to reach glycemic targets with a goal fasting glucose of 80 to 130 mg/dL without hypoglycemia. Prandial insulin was dosed according to postprandial glucose excursion. No other oral hypoglycemic agents were used in the population analyzed. The following data were retrospectively collected at baseline (when semaglutide was started) and after a 6-month follow-up period: age, sex, race, ethnicity, duration of diabetes, presence of comorbidities (diabetic retinopathy, neuropathy, diabetic kidney disease), height, weight, body mass index (BMI), fasting plasmatic glucose, glycated hemoglobin (HbA1c), total daily dose of insulin (TDD), C-peptide levels, metrics from continuous (CGM)/flash glucose monitoring, such as time in range (TIR), time above range, time below range, coefficient of variation, and glucose management indicator. The levels of C-peptide were measured in fasting conditions with recommendations to avoid fast insulin on the morning and slow release on the night before the C-peptide measurements.

### Statistical analysis

Quantitative data are described as mean ± SD and range, whereas qualitative variables are expressed as absolute number of cases and as percentage of the cohort of the evaluated patients. The normal distribution of all parameters was assessed by means of the Shapiro-Wilk test. Statistical differences were determined by Mann-Whitney *U* and Student *t*-tests for, respectively, nonparametric and parametric continuous variables. Discrete variables were compared by the χ^2^ test or the Fisher exact test. The statistical significance was defined as *P* < .05. Differences between groups were determined by ANOVA for normally distributed variables, and by the Mann-Whitney test for those not normally distributed. The levels of statistical significance were set at *P* < .05. All statistical analyses were performed using the statistical package SPSS for Windows version 20.0 (SPPS Inc., Chicago, IL). In cases of drop-out, only the data available till the time of drop-out were included in the analysis.

## Results

### Baseline characteristics of the study participants

A total of 80 patients with LADA who were treated with semaglutide as adjunct therapy to insulin were identified. LADA diagnosis was confirmed by age of onset, the presence of autoimmunity, and the slow progression towards insulin deficiency. Overall, data for 68 patients at baseline and at 6 months of follow-up were available, whereas 12 patients discontinued therapy. The main reasons for discontinuing semaglutide therapy were because of the gastrointestinal side effects (defined as any mention of nausea/vomiting, significant loss of appetite, significant diarrhea/constipation, abdominal pain, gastrointestinal acid reflux). The baseline and demographic characteristics of the 68 patients who completed the follow-up are shown in Table 1. The mean age was 47.8 ± 12.3 years, with 37 (54.4%) females and 31 (45.6%) males and a mean duration of diabetes of 8.8 ± 9.9 years. The baseline antidiabetic treatments consisted of basal insulin in 15/68 patients (22.1%) only and basal bolus insulin therapy in 53/68 (77.9%). All subjects started a treatment with semaglutide as follows: 11/68 patients received (16.2%) subcutaneous semaglutide at an initial dose of 0.25 mg/week, whereas most of the patients (57/68, 83.8%) started oral semaglutide at an initial dose of 3 mg/day. After initial titration, mean dosage of subcutaneous semaglutide was 0.80 ± 0.25 mg/week, while the mean dose of oral semaglutide was 9.2 ± 4.9 mg/day. Median HbA1c and median BMI at the start of the study were, respectively, 66.9 ± 19.3 mmol/mol (8.3 ± 1.7%) and 26.9 ± 6.4 kg/m^2^. C-peptide values were available for 49/68 subjects (72%); average C-peptide levels at baseline were 0.5 ± 0.4 nmol/L.

**Table 1:** Baseline characteristics of the study population

Parameters	Data
Age (years)	47.8 ± 1.5
Male (n)	31
Female (n)	37
Duration of disease (years)	8.8 ± 0.9
HbA1c (mmol/mol)	66.9 ± 2.4
HbA1c (%)	8.3 ± 0.2
FPG (mg/dL)	163.7 ± 15.2
C-peptide (nmol/L)	0.5 ± 0.1
S-creatinine (mg/dL)	0.88 ± 0.03
LDL-cholesterol (mg/dL)	93.4 ± 5.3
BMI (kg/m^2^)	26.9 ± 0.3
Normal weight (%)	50.1
Overweight (%)	29.4
Obesity (%)	20.5
Class 1	10.3
Class 2	2.9
Class 3	7.3
ACR (mg/gr)	25.9 ± 11.8
Micro-albuminuria (mg/L)	17.6 ± 1.8
Positive autoantibodies (%)	
GAD	95.4%
ZnT8	35.3%
ICA	60.5%
IA2	55.3%
Insulin	27.8%
Positive autoantibodies (%)	
Single	44.4%
Double	22.2%
Triple or more	12.7%

Data are expressed as mean ± SEM or n, %.

Abbreviations: ACR, albumin-creatinine ratio; BMI, body mass index; FPG, fasting plasma glucose; HbA1c, glycated hemoglobin.

### Semaglutide improved glycometabolic control in patients with LADA

**Figure 1:**
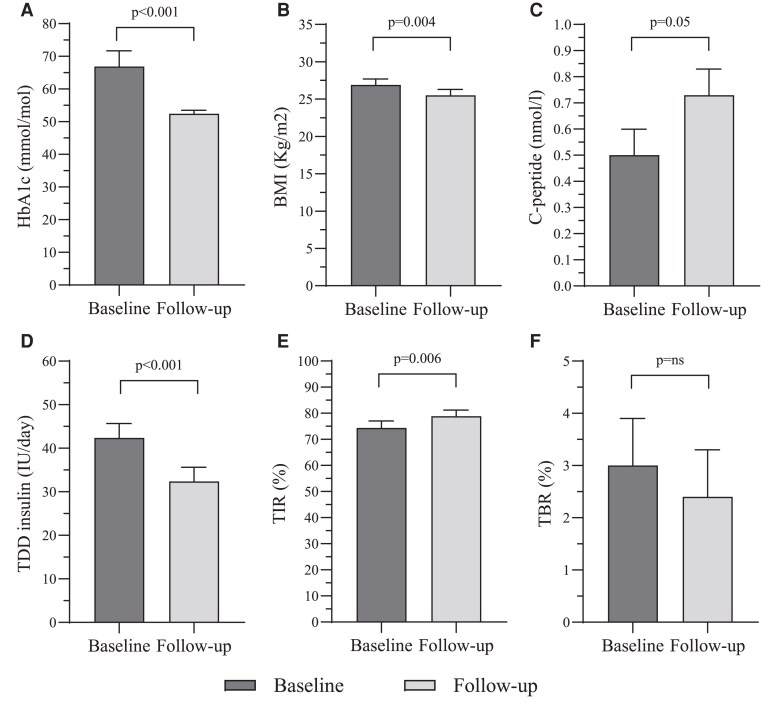
Changes from baseline to follow-up with semaglutide use in HbA1c (A), BMI (B), C-peptide (C), TDD (D), time in range (E), and time below range (F). Abbreviations: BMI, body mass index; HbA1c, glycated hemoglobin; TBR, time below range; TDD, total daily dose; TIR, time in range. *t*-test for repeated measures. Data are expressed as mean ± SEM.

We next assessed the effects of semaglutide treatment in our study population after a mean follow up of 6 months. Patients treated with semaglutide showed a reduction in HbA1c and BMI values compared to baseline (Table 2; Fig. 1). Importantly, there was an overall increase in C-peptide levels after 6 months of semaglutide therapy and a remarkable reduction in exogenous insulin requirement, with a striking reduction in insulin bolus dose. An improvement in glucose metrics was also observed as confirmed by the improvement in TIR values and the absence of any higher rate of hypoglycemia (Table 2; Fig. 1). Taken together, our data showed that the use of semaglutide as an adjunct therapy in patients with LADA was associated with an overall improvement of glycometabolic control.

**Table 2:** Changes in outcomes from baseline to follow-up with semaglutide use

	Baseline	Follow-up	*P* value
BMI (kg/m^2^)	26.9 ± 0.8	25.5 ± 0.7	.004
FPG (mg/dL)	163.7 ± 15.2	139.4 ± 3.6	.001
HbA1c (mmol/mol)	66.9 ± 2.4	52.4 ± 1.07	.001
HbA1c (%)	8.2 ± 0.2	6.9 ± 0.1	
C-peptide (nmol/L)	0.5 ± 0.1	0.73 ± 0.1	.05
TDD (IU/day)	42.4 ± 3.3	32.4 ± 3.2	.000
Basal insulin (IU/day)	20.5 ± 1.9	19.4 ± 1.65	Ns
Bolus insulin (IU/day)	21.8 ± 2.2	13.9 ± 2.3	<.001
TIR (%)	74.4 ± 2.6	78.9 ± 2.3	.006
TAR (%)	22.3 ± 2.6	17.5 ± 2.1	.003
TBR (%)	3.0 ± 1.0	2.4 ± 0.9	Ns
CV (%)	29.9 ± 2.26	28.2 ± 1.8	Ns
GMI (%)	6.9 ± 0.13	6.7 ± 0.1	Ns

*t*-test for repeated measures. Data are expressed as mean ± SEM or n, %.

Abbreviations: BMI, body mass index; CV, coefficient of variation; FPG, fasting plasma glucose; GMI, glucose management indicator; HbA1c, glycated hemoglobin; TAR, time above range; TBR, time below range; TDD, total daily dose; TIR, time in range.

### Semaglutide efficacy depends on the extent of β-cell reserve

**Figure 2:**
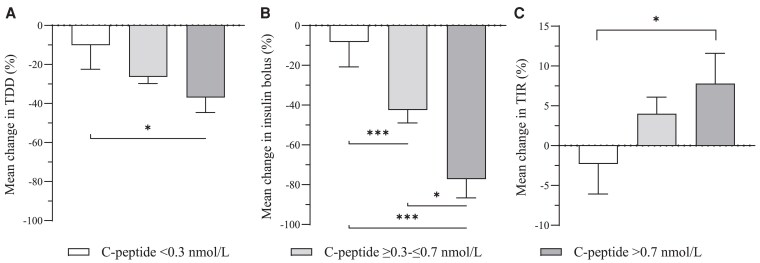
Percentage of mean change in TDD (A), bolus insulin dose (B), and time in range (C) at 6 months on semaglutide therapy, comparatively to the 3 baseline C-peptide clusters. Abbreviations: TDD, total daily dose; TIR, time in range. One-way ANOVA test. Data are expressed as mean ± SEM; *P < .05, ***P < .001.

To better understand the relevance of semaglutide therapy with respect to β-cell function, we stratified our study population according to their relative C-peptide values as follows: group #1: C-peptide <0.3; group #2: C-peptide ≥0.3 to ≤ 0.7; and group #3: C-peptide >0.7 nmol/L. The baseline characteristics and delta values at follow-up of the 3 groups are shown in Table 3; particularly significant differences were noticed regarding age and duration of the disease. An overall reduction of HbA1c, BMI, and total daily insulin dose was observed in all 3 groups, but it was more pronounced in those with preserved C-peptide values. Figure 2 shows the comparison between percentage of mean variations in the 3 groups at follow-up, regarding total daily dose, prandial insulin, and TIR values. As shown, our data demonstrated that the prandial insulin dose significantly decreased progressively at follow up, particularly in those patients with higher β-cell reserve (Fig. 2).

**Table 3:** Characteristics of the study population at baseline and mean delta at follow-up, according to β-cell function

	C-peptide <0.3 nmol/L (n = 7)	C-peptide ≥0.3-≤0.7 nmol/L (n = 32)	C-peptide >0.7 nmol/L (n = 10)	*P* value
Age (years)	63.3 ± 3	42.3 ± 2.2	47.3 ± 2.9	<.001
Duration of diabetes (years)	10.7 ± 3.0	4.6 ± 0.9	3.8 ± 0.8	.02
Gender (F/M)	6/1	13/19	6/4	Ns
BMI (kg/m^2^)				
Baseline	24.5 ± 1.3	24.4 ± 0.9	25.6 ± 1.6	Ns
Mean delta at follow-up	−0.3 ± 0.2	−0.9 ± 0.4	−0.9 ± 0.2	Ns
FPG (mg/dL)				
Baseline	156.3 ± 27.6	180.5 ± 18.6	167.2 ± 31.5	.001
Mean delta at follow-up	+4.3 ± 22	−34.6 ± 15.7	−46 ± 24.3	.001
HbA1c (mmol/mol, [%])				
Baseline	69.7 ± 9.7, [8.5 ± 0.9]	72.7 ± 3.5, [8.7 ± 0.3]	59.8 ± 5.6, [7.6 ± 0.5]	Ns
Mean delta at follow-up	−15 ± 9.7, [−1.3 ± 0.8]	−22.8 ± 3.4, [−2.0 ± 0.3]	−11.2 ± 4.8, [−1.0 ± 0.4]	Ns
TDD (IU/day)				
Baseline	28.6 ± 4.5	47.8 ± 3.6	31.3 ± 8.1	.02
Mean delta at follow-up	−2.9 ± 2.6	−12.6 ± 1.8	−11.6 ± 3.9	Ns
Basal insulin (IU/day)				
Baseline	14.1 ± 1.9	20.1 ± 1.3	15.5 ± 3.3	Ns
Mean delta at follow-up	−1.7 ± 1.8	−0.75 ± 0.6	−0.6 ± 0.3	Ns
Bolus insulin (IU/day)				
Baseline	14.4 ± 3.5	27.7 ± 2.8	15.8 ± 5.5	.03
Mean delta at follow-up	−1.2 ± 1.3	−11.8 ± 4.0	−12.2 ± 1.6	.03

One-way ANOVA test. Data are expressed as mean ± SEM.

Abbreviations: BMI, body mass index; F/M, female/male; FPG, fasting plasma glucose; HbA1c, glycated hemoglobin; TDD, total daily dose.

### Glucose metrics variations accordingly to β-cell reserve

**Figure 3:**
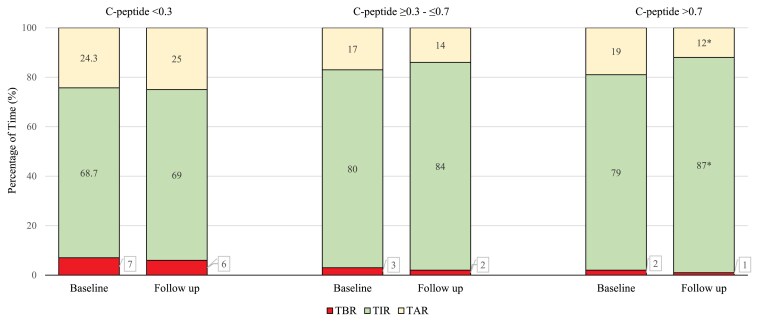
Changes in glucose metrics from baseline to follow-up, comparatively, to baseline C-peptide values. Abbreviations: TAR, time above range (>180 mg/dL); TBR, time below range (<70 mg/dL); TIR, time in range (70-180 mg/dL); *t*-test for repeated measures. Data are expressed as mean. *P < .05.

We then analyzed at follow-up the glucose metrics from the recorded CGMs in the 3 groups of patients (Fig. 3). As shown in Fig. 3, the subgroup of subjects with the most preserved β-cell mass (group #3: C-peptide > 0.7 nmol/L) had the strongest improvement in TIR values (+8%), whereas no differences in time below range were observed.

### Discontinuation of prandial insulin

Finally, considering the entire study population, we noticed a cessation of prandial insulin in 14 of 68 (20.5%) subjects treated with semaglutide. Notably, the majority of these patients (12/14) showed some preserved C-peptide values at baseline (>0.3 nmol/L). Patients who suspended insulin bolus administration were characterized by slightly younger age, although not statistically significant (suspender vs nonsuspender: 44.1 ± 11.5 vs 48.8 ± 12.2 years; *P* = ns), and by a shorter duration of diabetes (suspender vs nonsuspender: 4.4 ± 2.1 vs 7.3 ± 6.9 years; *P* = .01). Conversely, there were no differences regarding baseline BMI, HbA1c values, and baseline insulin dosage. Collectively, our findings suggest that semaglutide therapy was markedly beneficial in patients with LADA who maintained a preserved β-cell function.

## Discussion

In our study, we evaluated the short-term effects of semaglutide as an adjunct therapy to insulin in patients with LADA. Considering the entire studied population, semaglutide treatment led to an improvement of glycometabolic controls and to a significant reduction in total daily and bolus insulin use. Notably, the mean reduction in HbA1c (−14.5 mmol/mol, −1.3%) reported in our study was higher than what is reported by previous studies (−0.5%) (12, 13). However, most of the data from the literature investigated the efficacy of GLP-1R agonists as an adjunct treatment in patients with T1D whose C-peptide levels were almost undetectable (13, 14). This may explain in part the higher reduction in HbA1c observed in our patients with LADA, particularly in those with residual β-cell function and shorter duration of the disease. In fact, the post hoc analysis from the AWARD studies with the use of dulaglutide treatment also reported a similar decrease in HbA1c in, respectively, patients who are GADA-negative (−1.09%) as well as in those GADA-positive (−0.94%), and reported similar reduced efficacy of the therapy in patients LADA with scarce β-cell function (11, 15). A recent randomized, double-blind, crossover trial conducted in patients with T1D, to assess the efficacy of semaglutide treatment as an adjunct to Automated Insulin Delivery therapy with a shorter follow up (15 weeks), showed similarly more HbA1c decrease, particularly in subjects with detectable C-peptide levels (−0.7% vs −0.4%) (13). Another important finding of our study is the significant reduction in BMI (−1.4 kg/m^2^), which is concordant with data reported by previous studies in patients with T1D (12). Altogether, these data highlight the well-known beneficial effect of semaglutide on weight management and on the reduction in insulin requirement (16). Indeed, weight management is very relevant in patients with LADA, who often present higher BMI and features of metabolic syndrome (4, 6, 7), as confirmed by recent analyses from a large prospective study demonstrating worse metabolic control in patients affected by LADA compared to those with T2D, exposing the former to a higher risk of cardiovascular events (4). Semaglutide treatment was accompanied by a significant reduction in total daily insulin dose and a suspension of prandial insulin in 20.5% of subjects, particularly in those with preserved β-cell function. Our findings are comparable to those reported by similar trials on patients with T1D (12, 13, 17-19), which is due to the known insulin-sparing/beneficial effects with the use of GLP1-R agonists (20). Even higher rates of insulin suspension, both prandial and basal, were obtained in a population of early diagnosed T1D (21). Our results, extrapolated from the analysis of glucose metrics from CGM, showed an overall improvement in TIR in treated subjects, which reached +8% in those with C-peptide levels above 0.7 nmol/L, similar to what has been observed in other trials (13, 22). Indeed, a double-blind trial in adults with T1D using the Automated Insulin Delivery system showed a mean increase TIR of 8.8% in the subgroup treated with semaglutide as add-on therapy (22). Increased TIR is commonly associated with a lower risk of both microvascular and macrovascular complications (23) accompanied by a reduced risk of hypoglycemia, thus showing a meaningful achievement of a clinical target for glycemic control (24). The common and typical side effects for GLP-1R agonists were gastrointestinal disorders (25), which resulted in the discontinuation of the therapy in only 15% of the subjects, which is significantly lower than what is previously reported (12, 17). We did not record any cases of diabetic ketoacidosis during the observed period. GLP-1R agonists are generally considered safe regarding euglycemic diabetic ketoacidosis (DKA). However, there have been rare reports of DKA associated with their use (26, 27), particularly when combined with substantial insulin dose reductions and decreased food intake. The underlying mechanism may involve relative insulin deficiency and increased lipolysis, gluconeogenesis, and glycogenolysis pathways inhibited by the GLP-1R agonists. However, the evidence in the literature is still limited, especially for LADA patients, and additional studies are needed to confirm whether DKA is directly related to the application of GLP-1R agonists (28). Hence, the lack of any major safety concern, the improvement of glycometabolic controls and of C-peptide levels represent major strengths of semaglutide therapy that might be an attractive therapeutic option particularly for those with residual β-cell function due to the extreme heterogenicity of LADA and the narrow available therapeutic options (7, 10, 29, 30). Moreover, the anti-inflammatory effects of GLP-1R agonists may contribute to the maintenance of residual β-cell function (31, 32). Studies published have explored the role of GLP-1R agonists, on top of insulin treatment, in the preservation of C-peptide levels in T1D. Trials with exenatide and dulaglutide have reported a modest increase in plasma C-peptide levels after, respectively, 52 and 24 weeks of follow-up (33, 34), whereas other studies have shown that the addition of GLP-1RA does not prevent progressive C-peptide loss in T1D (35, 36). By contrast, GLP-1R agonists have been shown to improve plasma C-peptide levels in T2D (37), primarily by enhancing glucose-dependent insulin secretion, reducing glucotoxicity and insulin resistance. The intermediate clinical and metabolic features observed in LADA patients, could explain the improvement in C-peptide levels observed in our population. The main limitations of our study included the small sample size, albeit being one of the largest studies on the use of GLP-1R agonists in patients with LADA. Another confounding factor is the use of different semaglutide formulations, once-daily oral and once-weekly injectable, at different dosages. Semaglutide was administered at the maximum tolerated dose and the formulation chosen based on patient's preference. However, it is worth noting that a recent retrospective, real-world study, in a setting of T2D under routine care (38), showed that both formulations of semaglutide can be equally effective for glycometabolic control and weight management, particularly in the first 6 months of treatment. Larger investigational studies/trials may be needed to better define the role of semaglutide therapy in subjects with LADA; indeed, the lack of control group and the short duration of the follow-up (6 months) may be also considered when designing the next trial.

In conclusion, our study demonstrated that the use of semaglutide as an adjunct therapy in patients with LADA was associated with sustained beneficial effects on weight loss and on the glycometabolic controls, especially in those with preserved β-cell function.

## Data Availability

The datasets generated during and/or analyzed during the current study are not publicly available but are available from the corresponding author upon reasonable request.

## Abbreviations

BMI: body mass index
CGM: continuous glucose monitor
DKA: diabetic ketoacidosis
GADA: glutamic acid decarboxylase antibody
HbA1c: glycated hemoglobin
LADA: latent autoimmune diabetes in adults
T1D: type 1 diabetes
T2D: type 2 diabetes
TDD: total daily dose
T1R: time in range
